# A spatially adaptive synthetic vegetation index for monitoring ecosystem changes in climatically heterogeneous basins

**DOI:** 10.1038/s41598-026-54880-8

**Published:** 2026-06-08

**Authors:** Liqin Yue, Xinyan Liu, Nan Liang, Lei Yang

**Affiliations:** 1 Huanghe Science and Technology University, Zijingshan South Road, Zhengzhou, 450000 China; 2 Aerospace Information Research Institute, Henan Academy of Sciences, Chongshili, Zhengzhou, 450046 China; 3 Henan Academy of Sciences, Chongshili, Zhengzhou, 450046 China

**Keywords:** Synthetic vegetation index, Dynamic geographically weighted, Spatial heterogeneity, Vegetation monitoring, Ecology, Ecology, Environmental sciences

## Abstract

The vegetation in climatically heterogeneous regions exhibits significant spatial variability and temporal succession characteristics. It is crucial to obtain consistent vegetation characteristics in this region over time. Traditional single indices such as NDVI (Normalized Difference Vegetation Index), LAI (Leaf Area Index), and NPP (Net Primary Productivity) each have their own advantages, but they often show inconsistent trends when applied to complex vegetation. To effectively capture spatial heterogeneity and enhance the ecological interpretability, we propose a Dynamic Spatially Variable Weighted Synthesis Vegetation Index (DWS-SVI). Based on four Global Land Surface Satellite Dataset (GLASS) vegetation parameters (FVC (Fractional Vegetation Cover), LAI, NDVI, and NPP) and land cover types, this method employs the CRITIC method to perform dynamic weighting and generate continuous weight surfaces, ultimately synthesizing a comprehensive vegetation index at the pixel level. It combines “global trend and local adaptation” by integrating spatial heterogeneity modeling and multi-variable dynamic weighting, thereby overcoming the limitations of traditional methods in terms of spatial heterogeneity and ecological interpretability. Results show that over the past two decades, more than 69.4% of the area in the YRB has witnessed a significant improvement in vegetation conditions. The improvement was most notable in the summer, and it was mainly attributed to the improvement in the temperature and humidity conditions in this region. Compared with a single indicator, the DWS-SVI index can reflect the coordinated evolution of ecosystem structure and function, and can effectively suppress the observation errors caused by the bias of a single vegetation index, especially the “false greening” signals in transition zones and arid areas. Furthermore, the dominant factor map constructed based on DWS-SVI further reveals the differentiated driving mechanisms of ecosystems such as farmland, grassland, and forest, demonstrating that it has superior interpretability. This study provides a transferable framework for constructing spatially adaptive vegetation indices, enabling more reliable monitoring of ecosystem changes in large river basins and other climatically heterogeneous regions.

**Supplementary Information:**

The online version contains supplementary material available at 10.1038/s41598-026-54880-8.

## Introduction

Vegetation plays a fundamental role in regulating terrestrial carbon, water cycles, and maintaining biodiversity^1,2^. Long-term monitoring of vegetation dynamics is therefore essential for understanding ecosystem functionality, evaluating ecological restoration and informing climate adaptation policies. In large heterogeneous landscapes regions, vegetation dynamics are often controlled by interacting gradients of precipitation, temperature, topography, soil conditions, and human activities. These controls may vary substantially over space and time, making it difficult to obtain a consistent understanding of vegetation change from scattered field observations alone. Although ground observations provide valuable field evidence, their sparse spatial distributions, high costs, and limited temporal continuity capacity restrict their application in large heterogeneous landscapes^3,4^. Since the Advanced Very High Resolution Radiometer (AVHRR) provided global Normalized Difference Vegetation Index (NDVI) datasets in the 1980s, satellite remote sensing has become the primary tools for monitoring vegetation change over large regions.

A wide range of remote-sensing vegetation indicators has been developed to characterize different aspects of vegetation condition. Greenness-based indicators, such as NDVI and the Enhanced Vegetation Index (EVI), are widely used to capture photosynthetically active vegetation signals and have been applied extensively in studies of vegetation greening, browning, phenology, and drought response^5–7^ Structural indicators, such as the Leaf Area Index (LAI), provide information on canopy development and light interception, while Fractional Vegetation Cover (FVC) describes the proportion of the land surface covered by vegetation^6^. Productivity-related variables, such as Net Primary Productivity (NPP), further link vegetation monitoring to carbon uptake and ecosystem functioning^8^. Each indictor has its unique advantages in regional and global vegetation monitoring. However, inherent distinctions exist in the physical meanings and sensitivities among different indices, as a result, individual indicators may produce inconsistent signals in climatically heterogeneous landscapes. For instance, NDVI tends to saturate in high biomass regions and lacks sensitivity to non-photosynthetic components (e.g., litter), potentially underestimating biomass accumulation in forest canopies^9^. Although the Enhanced Vegetation Index (EVI) can mitigate the saturation, it is highly sensitive to atmospheric aerosol contamination^10,11^. LAI tends to be influenced by soil background in sparse vegetation areas, and FVC mainly reflects cover fraction and may be less sensitive to functional differences among vegetation types^12,13^. NPP provides productivity information but is affected by model structure, meteorological inputs, and parameterization uncertainty^8^. Ding et al. found that, during 2000–2015, 45.6% of global vegetated area experienced inconsistent trends in vegetation greenness, cover and productivity, especially in evergreen broadleaf forests, grasslands, open shrublands, woody savannas and croplands^14^.Zhou and colleagues also found large discrepancies among remote sensing indices for characterizing vegetation growth dynamics in Nepal^15^. Therefore, relying solely on a single vegetation index presents certain limitations for assessing the functionality of ecosystem.

To overcome the limitations of single indicators, multi-parameter synergy analysis has attracted attention in vegetation monitoring. Such approaches can combine information from different vegetation indicators, and therefore have the potential to provide a more comprehensive description of vegetation condition^16,17^. However, existing composite approaches still face several limitations. Most methods adopt fixed weights, simple average, or empirical aggregation schemes, which may implicitly assume that the relative contribution of each indicator is constant or can be represented by global statistical relationships. Such assumptions are often difficult to justify in heterogeneous landscapes regions, where vegetation processes differ among forests, croplands, grasslands, shrublands, wetlands, and sparsely vegetated areas^18,19^. Moreover, many composite indices are mainly driven by statistical aggregation, with limited ecological interpretation of indicator weights and insufficient representation of land-cover and climatic heterogeneity across ecological zones^20^. Therefore, a spatially and seasonally adaptive synthesis framework is needed to distinguish integrated vegetation-condition improvement from greenness-dominated changes caused by phenology, soil background, or short-term cover fluctuations.

Another important challenge is data consistency. Multi-indicator vegetation assessment requires variables that are comparable in space and time. However, differences in sensors, retrieval algorithms, preprocessing procedures, spatial and temporal resolutions, and gap-filling strategies can introduce additional uncertainty when different vegetation products are combined^21,22^. Long-term and internally consistent satellite products therefore provide an important basis for developing integrated vegetation-condition indicators. The Global LAnd Surface Satellite (GLASS) products provide long-term and spatially continuous vegetation index datasets derived from multi-source satellite observations under a relatively consistent algorithm framework. The availability of GLASS NDVI, LAI, FVC, and NPP makes it possible to construct an integrated vegetation-condition index that combines greenness, canopy structure, cover, and productivity using a more harmonized data foundation^23^.

The Yellow River Basin (YRB) is one of China’s most climatically and ecologically heterogeneous basins, spanning arid, semi-arid, and semi-humid regions and containing diverse land-cover types, including grasslands, croplands, forests, shrublands, wetlands, and sparsely vegetated areas^24^. In this basin, vegetation dynamics are jointly shaped by water availability, heat conditions, terrain, land-cover composition, ecological restoration, and human management. These characteristics make the YRB a challenging but representative case for evaluating whether a synthetic index can provide a more balanced assessment of vegetation condition than single indicators alone.

In this study, we developed a Synthetic Vegetation Index based on the concept of Dynamic Geographically Weighted (DWS-SVI, hereafter called SVI for simplicity). This index should not be interpreted as a new spectral vegetation index. Rather, it is a spatially adaptive synthesis framework that integrates four key vegetation indicators that represent the NDVI (greenness), LAI (canopy structure), FVC (cover), and NPP (productivity). However, unlike the traditional fixed-weight synthesis method, its weights can be dynamically adjusted according to land cover type, seasonal changes, and spatial location. By integrating spatial heterogeneity modeling with dynamic multivariate weighting mechanisms, SVI not only coordinates the greenness, structure, and functional characteristics of vegetation, but also adapts to seasonal changes and ecosystem heterogeneity. This enhances ecological interpretability and transferability across regions. We aim to (1) assess the spatial and temporal patterns of vegetation dynamic changes from 2000 to 2021, (2) evaluate the consistency and divergence between SVI and individual vegetation indicators, and (3) examine the robustness of SVI and the underlying ecological driving mechanisms. This study provides a new method and perspective for promoting remote sensing vegetation monitoring in large river basins and other climatically heterogeneous regions.

## Materials and methods

### Study area

**Fig. 1:**
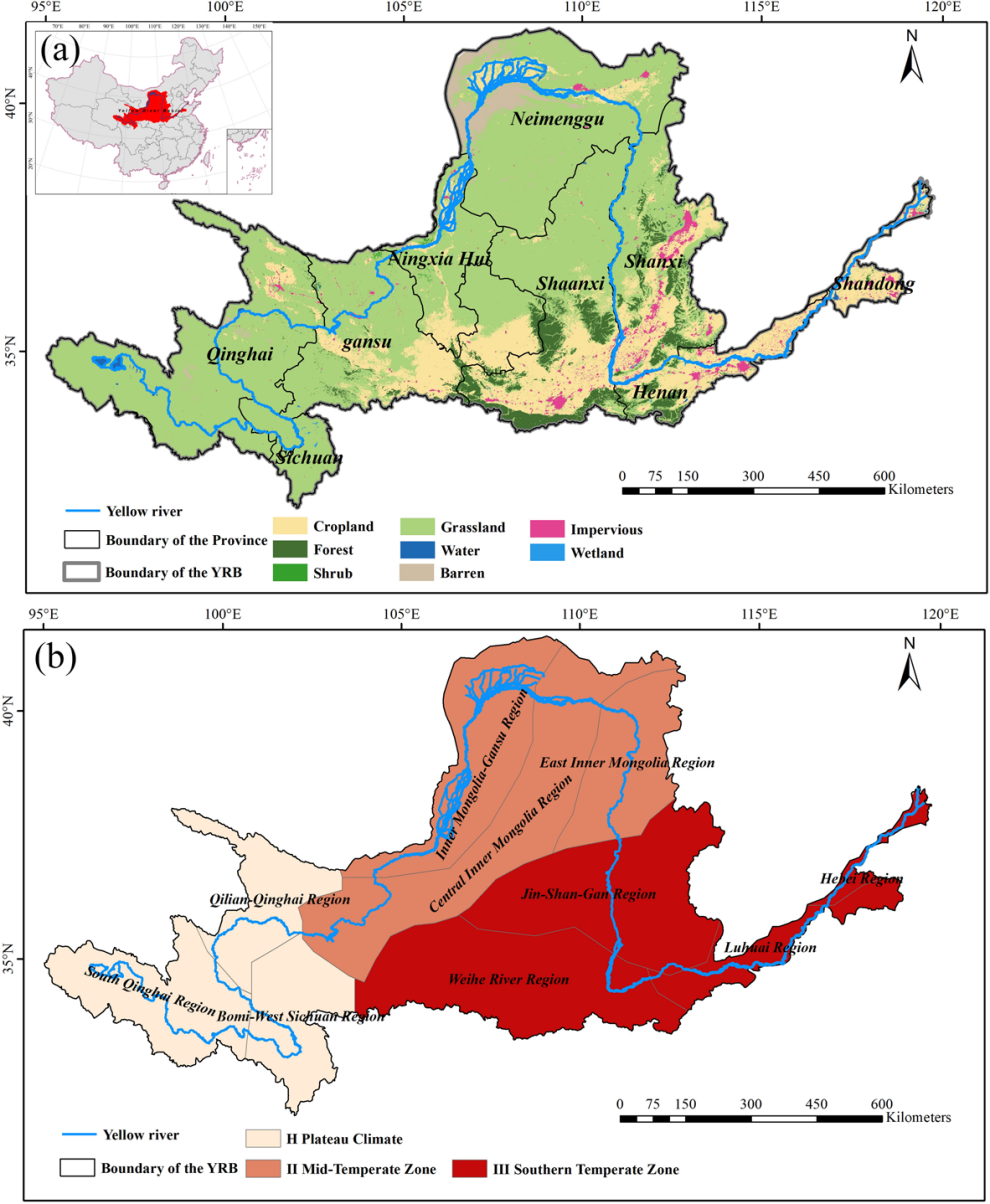
The geographic location and overview of the Yellow River Basin (YRB). (**a**) the spatial distribution of landcover in the YRB, and (**b**) the climate division of the YRB.

The YRB is located in northern China (30° N–42° N, 94° E–120° E; Fig. 1a), traversing diverse geomorphological units such as the Tibetan Plateau, Loess Plateau, Inner Mongolia Plateau, and the Huang-Huai-Hai Plain, covering a total area of 795,000 km^2^^25^. The climate division indicates a significant spatial gradient across the YRB^26^. The “H Plateau Climate Region” has an annual accumulated temperature below 2000 °C, spans arid to semi‑arid zones, and is generally unsuitable for forest growth. The “II Mid‑Temperate Zone” (accumulated temperature 1600–3400 °C) experiences severe winters and lies within arid to semi‑arid areas. The “III Warm‑Temperate Zone” (accumulated temperature 3400–4500 °C) is characterized by cold winters and hot summers, extending across humid to semi‑humid regions (Fig. 1b). This climate division is sourced from the Resource and Environmental Science Data Platform (https://www.resdc.cn/data.aspx?DATAID=243), originally compiled by the China Meteorological Administration in 1978 based on 1951–1970 records. The land-cover types are also diverse, including high-altitude grasslands and deserts in the upper reaches, as well as croplands and forests in the middle and lower reaches^27^. These strong climatic and ecological gradients, make the YRB a suitable case for evaluating a spatially adaptive vegetation-monitoring method under complex surface conditions.

### Data sources and preprocessing

The Global Land Surface Satellite (GLASS, https://glass.hku.hk) products are long-term, high-resolution, and high-accuracy satellite-derived datasets. They are derived by integrating multi-source remote sensing observations with ground-based measurements using consistent inversion algorithms, which provides seamless records and minimizes cross-sensor discrepancies^28^. In this study, we employed four GLASS vegetation parameters, including NDVI, LAI, FVC, and NPP, spanning from 2000 to 2021. These four indicators are ecologically complementary, capturing greenness (NDVI), canopy structure (LAI), coverage (FVC), and ecosystem function (NPP) respectively, thereby providing a more comprehensive reflection of the overall growth status of the vegetation.

The GLASS LAI product (version 6) is developed using the bidirectional long short-term memory (Bi-LSTM) deep learning model driven by MODIS surface reflectance^29^. It is available at 250 m and 500 m spatial resolutions, with 8-day and monthly temporal steps. Compared with other global LAI products, GLASS LAI V6 shows superior temporal continuity, reduces cloud/snow contamination, and improves accuracy compared to field measurements^30–32^. GLASS NDVI is reconstructed using an LSTM neural network which was trained by the Savitzky-Golay filter, GLASS LAI fitting, and upper envelope methods^33^. This approach effectively mitigates cloud noise and data gaps, yielding spatially consistent and temporally continuous NDVI series at 250 m resolution. As NDVI is susceptible to the influence of soil and atmosphere, we mask the values below zero to avoid contamination from bare soil, snow, and water surfaces. GLASS FVC is generated using the MARS (Multivariate Adaptive Regression Splines) method^34^. The validation using ground measurements and high-resolution satellite data indicates that this data can obtain reliable vegetation coverage in information on both regional and global scales^35,36^. GLASS NPP product is generated by integrating GLASS GPP data with simulations from ten dynamic vegetation models in the TRENDY project^37^. It explains the differences in light energy utilization efficiency between autotrophic respiration, light exposed and shade protected leaves, as well as the effects of drought and CO₂ fertilization. This design provides ecologically meaningful measures of carbon sequestration capacity, directly linking vegetation monitoring to ecosystem productivity and functionality.

The land cover classes came from MODIS Land Cover Type Product (MCD12Q1) (https://lpdaac.usgs.gov/products/mcd12q1v006, accessed on 10 May 2025). To ensure that the weighting scheme can reflect interannual land cover changes, we used the annual land cover classification data from 2000 to 2021. In addition, meteorological data were used to provide contextual understanding of the environmental background of the YRB. Specifically, monthly mean temperature and monthly precipitation data from 2001 to 2021 were sourced from the Climatic Research Unit Time-Series (CRU TS v4.09) dataset provided by the National Centre for Atmospheric Sciences (NCAS), UK (https://crudata.uea.ac.uk/cru/data/hrg/, accessed on 10 May 2025). The CRU TS dataset is one of the most widely used climate datasets globally, offering temperature and precipitation data at a 0.5° spatial resolution over all land areas (excluding Antarctica) from 1901 to the present^38^.

### Methods

#### Construction of the SVI

The dynamic spatially variable weighted synthesis (DWS) method framework integrates four key indicators closely related to vegetation greenness, canopy structure, vegetation cover, and productivity to construct a spatially and temporally heterogeneous synthetic vegetation index. This method dynamically adjusts the weights based on the local environment and vegetation conditions, effectively overcoming the limitations of traditional methods in handling spatial heterogeneity and ecological interpretability.

1. Normalization of Vegetation Indices

To ensure the comparability across vegetation parameters with different units, a min-max normalization is applied to each vegetation parameter prior to index construction, scaling all values to the [0, 1] range:

1 $$\:N=\left(VI-{VI}_{min}\right)/\left({VI}_{max}-{VI}_{min}\right)$$

where *N* denotes the normalized value, *VI* represents the vegetation parameter for a given pixel, and *VI*_*min*_ and *VI*_*max*_ are the minimum and maximum values for that parameter, respectively.

This study divided the YRB into eight land cover types according to the annual land cover classification results of MCD12Q1(Fig. 1a). The classification originates from the International Geosphere-Biosphere Programme (IGBP), which includes eighteen classes. To enhance calculation efficiency, we categorized the IGBP vegetation classes into cropland, forest, shrubland and grassland based on vegetation types, and defined four non-vegetation classes (water, barren land, impervious surfaces, and wetland). These eight land cover types are used as the basic units for weight calculation. To reduce the influence of temporal and spatial heterogeneity on weight determination, for each year, we extracted the vegetation parameter data within each land cover type and each month, and constructed a three-dimensional data matrix organized by land cover type and month. This method effectively ensures that the weight scheme can simultaneously take into account the temporal change characteristics and land cover heterogeneity.

(2) Calculation of Adaptive Weights

Instead of assigning fixed global weights, we employ the CRITIC (CRiteria Importance Through Intercriteria Correlation) method^39^ to objectively determine the weights for the four indicators (NDVI, LAI, FVC, and NPP) for each year, each land cover type and each season. Unlike traditional fixed weighting methods, this approach enables the weights to be adaptively adjusted according to the dynamic changes of local vegetation and the redundancy among indicators, thereby enhancing the ecological interpretability. The specific calculation process includes four steps. Firstly, calculating the standard deviation of each parameter across all pixels within that land cover type and season to quantify its variability. A higher standard deviation indicates that the parameter captures a greater range of variability across the study area, and thus should contribute more to the integrated index. Then the absolute correlation matrix among parameters was computed to assess redundancy. The higher the correlation between an indicator and other parameters, the greater the degree of information overlap, and the corresponding weight should be reduced. The third step is to determine the information content of each parameter based on its variability and redundancy. Parameters with high variability and low redundancy are considered to have a greater impact on the comprehensive evaluation system and should be assigned higher weights. Finally, the final objective weights for each parameter were calculated based on the amount of information.

2 $$\:{\omega\:}_{j}=\frac{{I}_{j}}{\sum_{k=1}^{m}{I}_{k}}$$

3 $$\:{I}_{j}={\sigma\:}_{j}\sum\limits_{i=1}^{m}(1-{r}_{ij})$$

where *ω*_*j*_ denotes the normalized objective weight of parameter *j*. *I*_*j*_ represents the information of parameter j. *σ*_*j*_ is the standard deviation, which represents the internal contrast intensity of parameter j. *r*_*ij*_ is the correlation coefficient between parameters *i* and *j*. *m* represents the total number of individual vegetation indicators, and *k* is a dummy variable used during the summation process.

(3) Construction of Continuous Weight Surfaces

To capture fine-scale spatial heterogeneity, the weights calculated each year based on discrete land cover types were generated as a continuous surface through interpolation methods. For each land cover type, 1,000 random sampling points were selected each year (if the number of valid pixels is less than 1000, all were extracted), and their geographical coordinates and parameter weight values were recorded. Based on the sampling weight data of each vegetation parameter, a spatial continuous surface was constructed using the cubic spline interpolation method, and finally, the parameter weight distribution maps for each month of the entire watershed were generated. This processing procedure converts the discrete weights based on land cover types into dynamic and spatially position-adaptive continuous weights, avoiding weight jumps at type boundaries and accurately reflecting the gradually changing ecological gradient characteristics within the watershed.

(4) Construction of the Synthetic Vegetation Index

Finally, the DWS-SVI was calculated at the pixel level by integrating normalized vegetation parameters with dynamically varying weights. For a given year *y*, month *m*, and location (*lat*, *lon*), the index was computed as:

4 $$\:SVI\left(y,m,lat,lon\right)=\sum\limits_{j=1}^{m}{\omega\:}_{j}\left(lat,lon\right){N}_{j}\left(y,m,lat,lon\right)$$

where *ω*_*j*_ (*lat*, *lon*) is the spatially interpolated weight for parameter *j*, and *N*_*j*_(*y*, *m*, *lat*, *lon*) is the normalized vegetation parameter.

**Fig. 2:**
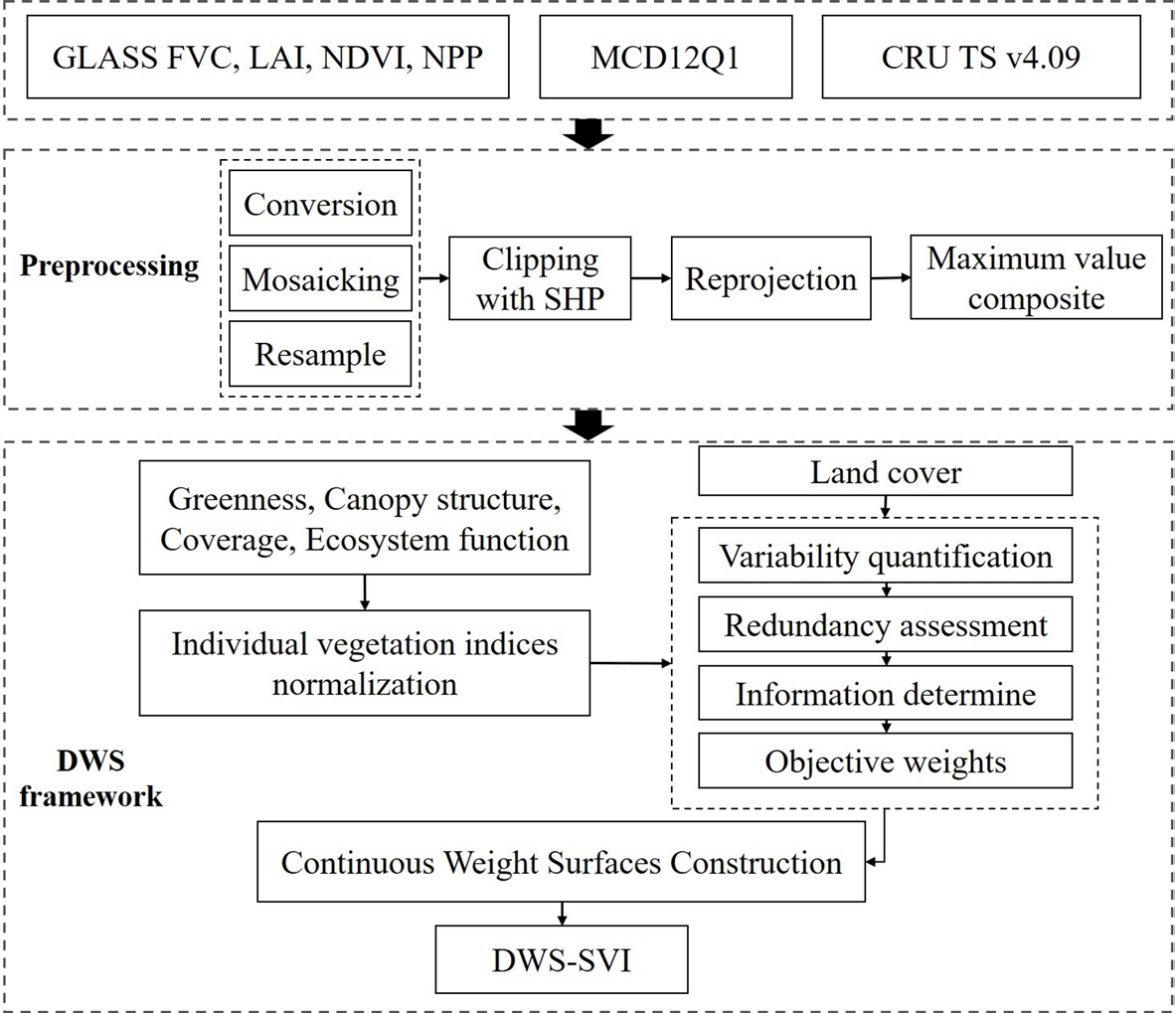
Flowchart of the dynamic spatially variable weighted synthesis (DWS) framework.

Unlike traditional fixed-weight methods, DWS-SVI enables the relative contribution degree of NDVI, LAI, FVC, and NPP to vary dynamically spatially and seasonally. For example, NDVI may dominate in sparsely vegetated regions where greenness is the most sensitive indicator, while NPP or LAI may contribute more in high-productivity ecosystems. This spatial adaptivity not only improves the mathematical robustness of the index but also enhances its ecological interpretability, as the weights reflect the dominant vegetation characteristics in different ecological regions. The flowchart of the proposed framework is showed in Fig. 2.

#### Spatiotemporal analysis methods

To quantify monotonic trends in vegetation indices over time, we adopted the Theil–Sen statistics method^40,41^. It can be used to determine whether a trend exists in time-series data, as well as the direction and strength of the trend but resistant to the influence of missing values and the distributional form. Its greatest advantage is insensitivity to outliers and does not require the data to follow normal distribution. The Theil–Sen trend estimation equation is:

5 $$\:\beta\:=mean\left(\frac{{x}_{j}-{x}_{i}}{j-i}\right),\:\forall\:j>i,$$

where *x*_*i*_ and *x*_*j*_ denote the *i*-th and *j*-th values of the timeseries *x*_*k*_, *β* denotes the slope, and mean denotes the median of all slopes. When *β* > 0, an increasing trend is indicated; conversely, a decreasing trend is indicated.

The Mann–Kendall (M–K) test can be used to examine the significance level of trends in time-series data. In the M–K test^42^, for any independent and identically distributed random samples *x*_*i*_ (*i* = 1,2,……,*n*; *i*  <  *j *< *n*), under a two-sided test, the test statistic is:

6 $$\:R=\sum\limits_{i=1}^{n-1}\sum\limits_{j=i+1}^{n}sgn\left({x}_{j}-{x}_{i}\right),$$

where,

7 $${sgn} \left( {x_{j} - x_{i} } \right) = \left\{ {\begin{array}{*{20}l} {1\left( {x_{j} - x_{i} } \right) > 0} \hfill \\ {0\left( {x_{j} - x_{i} } \right) = 0} \hfill \\ { - 1\left( {x_{j} - x_{i} } \right) < 0} \hfill \\ \end{array} } \right\},$$

*R* is the sum of the step-function values of the differences between any two distinct points in the series. If *R* > 0, the observations in the later part tend to be larger than those in the earlier part; Conversely, if *R* < 0, the later observations tend to be smaller. *R* follows a normal distribution with mean 0. Its standard deviation can be expressed as:

8 $$\:Var\left(R\right)=n\left(n-1\right)\left(2n+5\right)/18,$$

When *n* > 10, the standardized normal variable is computed as:

9 $$Z = \left| {\begin{array}{*{20}l} {\left( {R - 1} \right)/\sqrt {Var\left( R \right)} } \hfill & {R > 0} \hfill \\ 0 \hfill & {R = 0} \hfill \\ {\left( {R + 1} \right)/\sqrt {Var\left( R \right)} } \hfill & {R < 0} \hfill \\ \end{array} } \right|,$$

In a two-sided trend test with a given confidence level α, if |*Z*|≥*Z*_*1-α/2*_, the null hypothesis is rejected, indicating that the time series data shows a significant increasing or decreasing trend. A positive Z indicates an increasing trend, and a negative *Z* indicates a decreasing trend; |*Z*| greater than or equal to 1.645, 1.96, and 2.576 indicates that the test passes the significance levels of 90%, 95%, and 99%, respectively. In this study, we performed the M-K trend test on the spatial trends of different indices and classified the changes of each index into seven trend types: Decrease++ (*P* < 0.01), Decrease+ (*P* < 0.05), Decrease (*P* < 0.1), Increase++ (*P* < 0.01), Increase+ (*P* < 0.05), Increase (*P* < 0.1), Insignificant change.

## Results

### Spatial distribution of individual vegetation indices

**Fig. 3:**
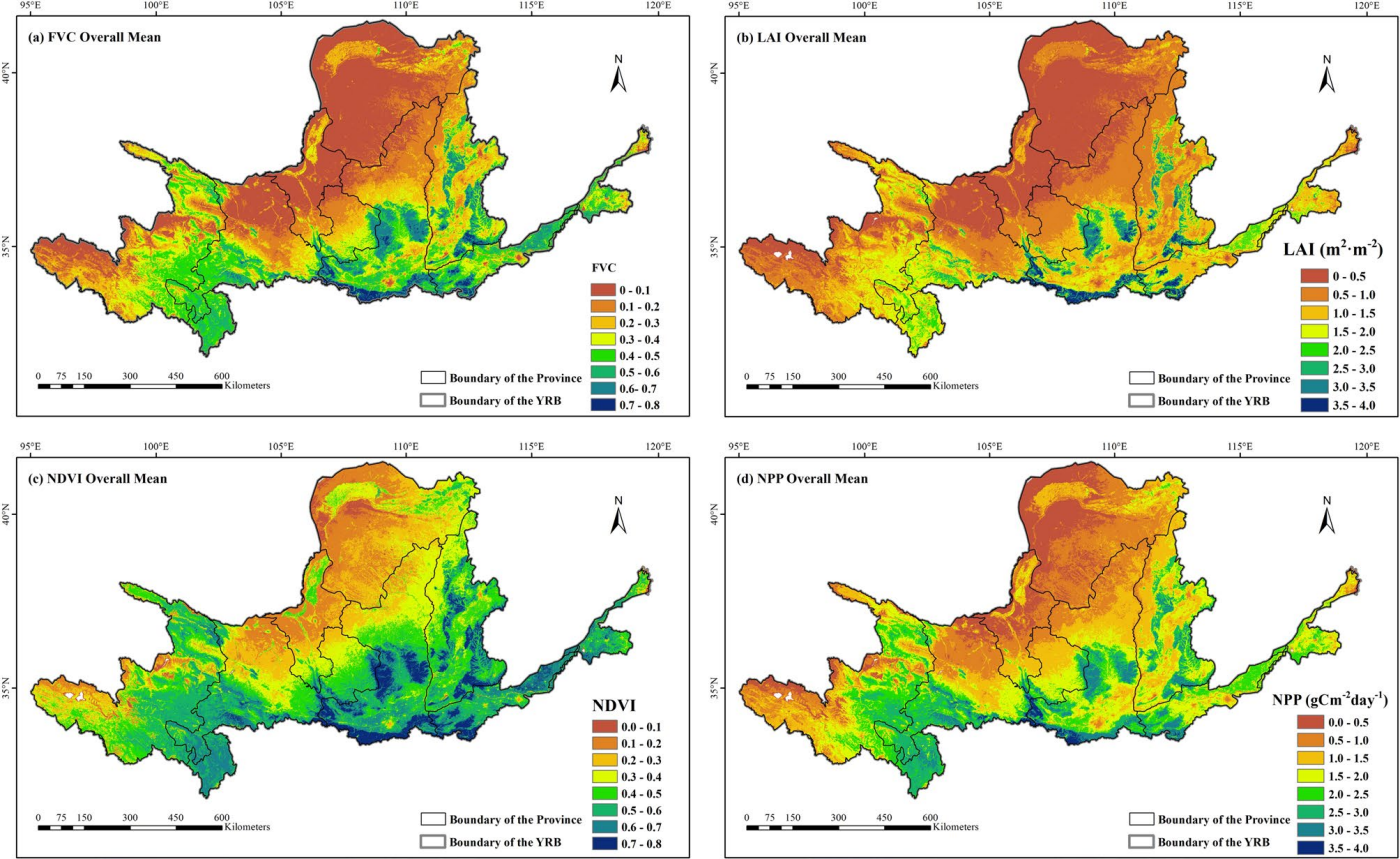
The spatial distribution of vegetation indices in yellow river basin.

Figure 3 shows the spatial distribution of the annual mean vegetation index in the YRB form 2000–2021, revealing spatial differences in the vegetation growth conditions represented by different indices. To reduce the uncertainty caused by the unstable winter vegetation signals, this study focuses on the vegetation parameters during the growing season (March to November). Overall, NDVI, LAI, FVC, and NPP show a consistent large-scale distribution pattern across the entire basin, reflecting the regional climate and topographic gradient characteristics. The high value areas of all indices are concentrated in the humid southeastern and middle-lower regions, while the arid northwest and high-altitude source areas show significant low values. This distribution pattern is closely related to the regional water and heat conditions, topographic features, and human land use activities.

The middle and lower reaches of the YRB (including Shandong, Henan, southern Shanxi and the Guanzhong Plain of Shaanxi) is located in the warm temperate semi-humid zone, with a mild climate. The average temperature during the growing season is approximately 18–20°C, and the annual precipitation exceeds 600 mm, with significant seasonal variations (see Figure A1). The area is characterized by the intermingling of forests, farmlands and grasslands, and the agricultural production is highly intensive. The average FVC of the forest ecosystem during the multi-year growing season is usually greater than 0.6, the average LAI is greater than 3.0 m^2^ m^−2^, the average NDVI is greater than 0.7, and the average NPP is greater than 3.0 g C m^−2^ d^−1^. These indicators present stable greenness, canopy structure, surface coverage and productivity characteristics. In contrast, the values of various indicators in the farmland are generally lower. In the monsoon fringe areas such as southern Gansu and most of Shanxi Province, the farmland FVC is approximately 0.2–0.4. While in southern Shaanxi, Henan and Shandong, the farmland FVC is slightly higher (about 0.4–0.5), which is closely related to more abundant and stable monsoon precipitation, good soil water storage conditions in the plain areas, and a relatively complete irrigation system. The distribution patterns of LAI, NDVI and NPP are highly consistent with FVC: in the southern part of Gansu Province and in Shanxi region, LAI is approximately 0.5–1.5 m^2^ m^−2^, NDVI is 0.3–0.5, and NPP is about 1.5–2.0 g C m^−2^ d^−1^; in Shaanxi, Henan and Shandong regions, the values of each indicator are relatively higher. This distribution feature highlights the synergistic improvement effect of water conditions improvement and agricultural management investment on the canopy structure and productivity.

The middle reaches of the YRB (covering the Inner Mongolia Plateau, northern Ningxia, the Loess Plateau of northern Shaanxi, and some areas of Gansu) is located in the semi-arid zone of mid-temperate zone. This area has scarce but relatively uniform precipitation, with the vegetation mainly consisting of grasslands, shrublands and deserts. Land degradation and wind erosion problems are prominent. The four vegetation indicators are generally low here: FVC < 0.3, LAI < 1.0 m^2^ m^−2^, NDVI < 0.4, and NPP < 1.5 g C m^−2^ d^−1^. These values reflect the strong limitation of water conditions on vegetation density and productivity under the combined effects of weakened monsoon influence, continuously high potential evaporation, and insufficient soil water supply, resulting in a relatively high ecosystem vulnerability. In contrast, the irrigation agricultural areas distributed along the main river course (such as the Ningxia Plain and the Hetao Plain) have benefited from the irrigation provided by the Yellow River and the temporal and spatial redistribution of water and heat resources in the river valleys. As a result, this region exhibits higher vegetation coverage, crown development degree, greenness, and productivity, forming a typical “green corridor” effect.

The upper reaches of the YRB lie in a high-latitude, and semi-arid region, with a relatively mild climate but low precipitation (Figure A1). The annual precipitation is moderate but unevenly distributed. The vegetation is mainly grassland and desert. The vegetation indicators show a significant two-level pattern: in the high-altitude and low-temperature area in the western part of Qinghai and the inland arid zone, the summer monsoon is difficult to penetrate and there is limited interannual moisture increase. All vegetation indicators are at a low level (FVC < 0.2, LAI < 1.0 m^2^ m^−2^, NDVI < 0.4, NPP < 1.5 g C m^−2^ d^−1^), indicating that low temperature and drought have a dual inhibitory effect on the alpine vegetation, resulting in sparse coverage, weak canopy structure and slow biomass accumulation. In the southwestern part of Gansu Province, the southeastern part of Qinghai Province, and some areas of Sichuan, the superimposed effect of the topographic uplift along the eastern edge of the Qinghai-Xizang Plateau and the water vapor transport from the southwest results in a precipitation of around 700 mm during the growing season (see Figure A1). Coupled with a warming trend of more than 0.5 °C every decade, the vegetation indicators have significantly improved: FVC reaches 0.4–0.7, LAI is 1.5–3.0 m^2^ m^−2^, NDVI reaches 0.5–0.7, and NPP reaches 2.0–3.5 g C m^−2^ d^−1^. This highlights the strong support of the water and heat coupling conditions for the structure and function of the mountainous ecosystem on the Qinghai-Xizang Plateau.

**Fig. 4:**
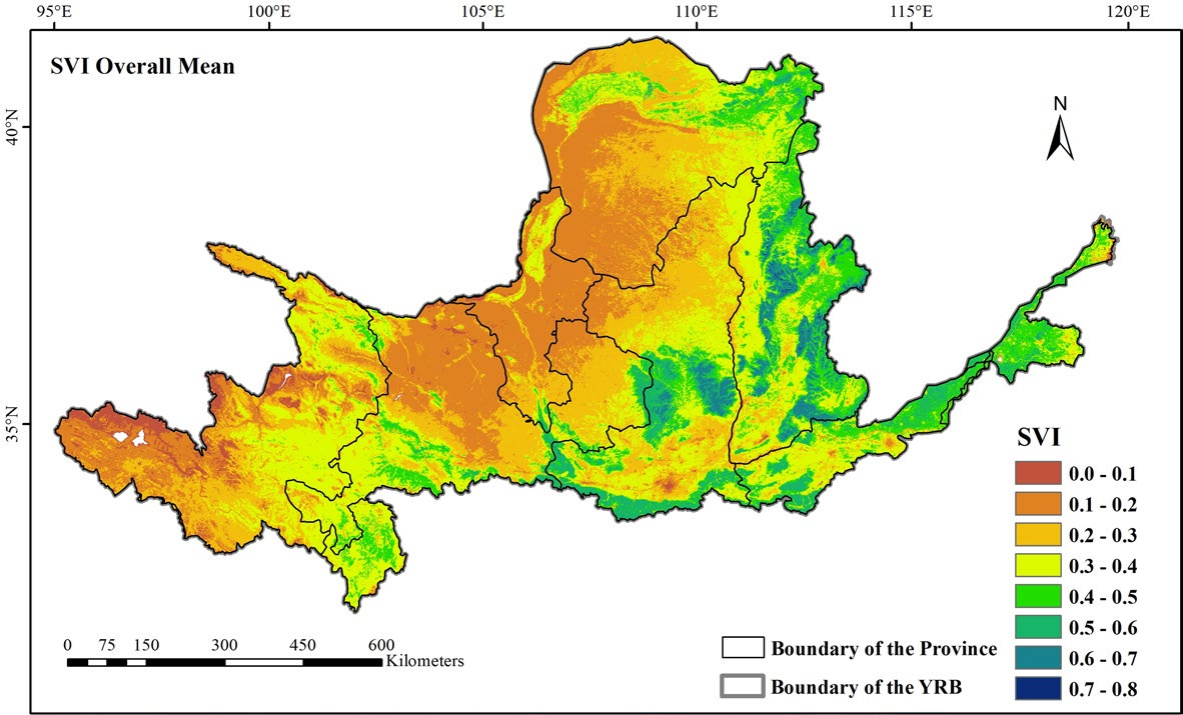
The spatial distribution of SVI in yellow river basin.

To comprehensively assess the growth status of vegetation, we systematically compared the spatial consistency and divergence between the SVI and four individual indicators (NDVI, FVC, LAI, NPP). As shown in Fig. 4, at the basin scale, SVI aligns well with individual indicator. In areas with favorable vegetation conditions (such as the downstream agricultural areas, the southern mountainous forests, and the eastern hilly regions), the SVI is generally above 0.5. However, in ecologically fragile areas (such as the northwest Loess Plateau, the western part of the Ordos Plateau, and the Hexi Corridor), SVI falls below 0.3. This distribution pattern conforms to the dominant rule revealed by individual indicators, namely “higher in the south and lower in the north, stronger in the east and weaker in the west”, and confirms the sensitivity of SVI to changes in surface greenness and vegetation coverage. Moreover, in regions with significant gradients in terrain and climate (such as the Qinling-Huangtu Plateau transition zone), the changes in SVI, LAI, and NPP show a highly consistent trend, effectively capturing the structural transformation characteristics of forest-grassland-pasture, and demonstrating the ability of SVI to depict the continuous spectrum of ecosystem functions along the water and heat gradients.

While maintaining this overall consistency, SVI exhibits lower variability in its differences than any single index. This is mainly due to the regulatory and identifying effects brought about by its multi-source data fusion. In areas with dominant crops (such as the Yellow River Delta, southwestern Shandong, and eastern Henan Plain), NDVI and FVC often show high values due to the seasonal phenology of crops, while LAI and NPP are relatively low, reflecting the complexity of the ecosystem structure. At this time, SVI presents a moderately low level (0.3–0.5), avoiding the overestimation of functions caused by relying solely on greenness. In the western part of the Loess Plateau and the Hexi Corridor, LAI is often relatively low because canopies are sparse or low-stature. However, the NDVI, FVC, and NPP are locally higher, indicating considerable vegetation density and production potential in those areas. In such scenarios, SVI tends to show higher values (> 0.5), demonstrating its ability to integrate structural and functional dimensions and correcting the potential deficiency caused by relying solely on LAI. Additionally, in transitional zones (such as the edge of the Loess Plateau, the Hexi Corridor, and the Inner Mongolia grassland), NDVI may occasionally reach a peak during occasional weather events (such as heavy rainfall) in summer. But due to the limited total and incremental precipitation, insufficient root-zone moisture inhibits leaf expansion, resulting in relatively lower LAI and FVC. In these areas, SVI presents a low-value pattern (typically 0.2–0.4), effectively suppressing the uncertainty caused by fluctuations in a single index.

**Fig. 5:**
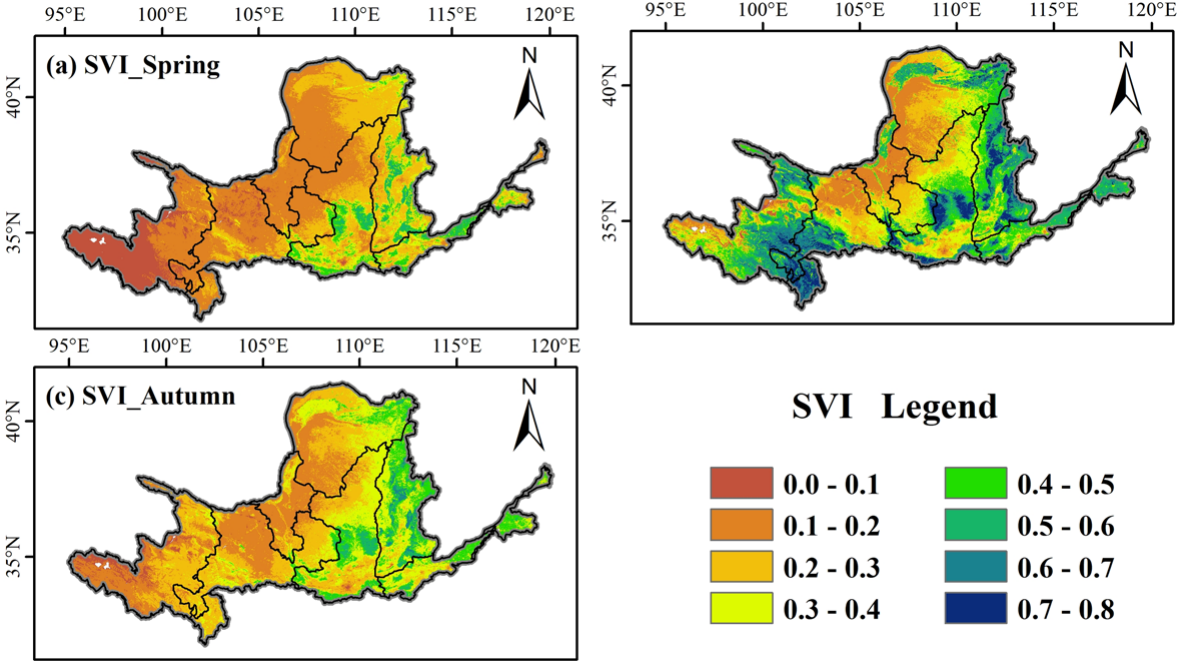
Spatial distribution characteristics of the synthetical vegetation index (SVI) in (**a**) spring, (**b**) summer, and (**c**) autumn.

Figure 5 shows that the vegetation in the YRB exhibits significant intra-seasonal sequence changes during the growing season (spring, summer, autumn), following a typical unimodal trajectory from green-up to peak growth and then senescence. In spring (March–May), limited by relatively low temperatures and soil moisture storage (Fig. A2), vegetation is generally at the early green-up stage, and SVI values are widely low—below 0.3 over most areas. Particularly on the Loess Plateau, in the Hexi Corridor, and along the eastern margin of the Tibetan Plateau, SVI commonly falls below 0.1, indicating that large-scale growth has not yet initiated. Because the increasing temperature and precipitation from the northwest toward the southeast, the lower reaches of the YRB (such as eastern Henan and southwestern Shandong) benefit from the simultaneous improvement of water and heat conditions, facilitating the green-up of winter wheat and the emergence of spring-sown crops, with SVI slightly higher and locally exceeding 0.3. In summer (June to August), the temperatures across the entire basin are generally within the suitable range of 25–30 °C, with enhanced precipitation, forming a high precipitation zone (about 80–200 millimeters, Figure A3) in the east and the southern edge. At this time, croplands and broadleaf vegetation reach their growth peak, and SVI increases markedly, commonly exceeding 0.4. The Huanghuaihai Plain, Guanzhong Basin, and the Yellow River Delta emerge as high-value centers, with local SVI greater than 0.7, consistent with the optimal water and heat condition in summer. During the late growth period (September–November), as temperatures decline and crops mature, the SVI generally decreased. However, the rates of spatial decline are uneven. The eastern forest and the agricultural areas receive more precipitation (20–50 millimeters) and relatively mild temperatures (8–15 °C, Figure A4), and the degree of artificial planting and irrigation is high. Therefore, the vegetation activity is high, keeping the SVI at approximately 0.3 or above. By contrast, in the western Loess Plateau, parts of the irrigation corridor along the mainstem, and the southwestern mountains, earlier leaf-yellowing and senescence drive SVI rapidly below 0.2, indicating a significant weakening of growth activities.

### Temporal trends of individual vegetation indices

#### Annual trend

**Fig. 6:**
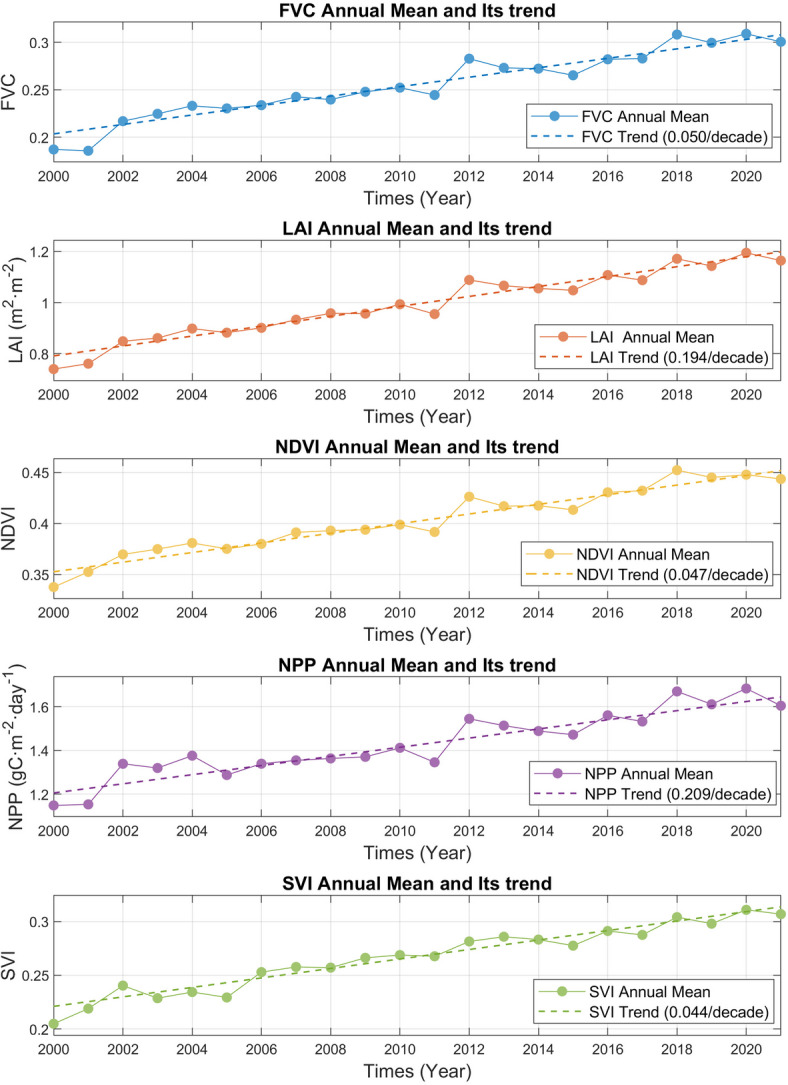
Annual mean and trends of different vegetation indices in yellow river basin.

To explore the temporal evolution characteristics of vegetation growth in the YRB, we calculated the annual means of multiple vegetation indices during the growing-season from 2000 to 2021. As shown in Fig. 6, all indices exhibit significant upward trends during the study period, indicating that the vegetation conditions in the YRB continued to improve. The mean FVC increased from 0.18 in 2000 to 0.30 in 2021, with a trend of approximately 0.05 per decade. LAI and NDVI also rose markedly at 0.194 m^2^ m^−2^ per decade and 0.047 per decade, respectively. As a proxy for productivity and carbon-sequestration capacity, NPP increased at ~ 0.209 gC m^−2^day^−1^ per decade, pointing to strengthened energy storage and ecosystem carbon uptake in YRB over the past two decades. During the same period, SVI increased from ~ 0.21 to ~ 0.31 with a slope of 0.044 per decade, demonstrating a highly consistent positive trends with each individual vegetation index. All the trend tests of the individual indices passed the Mann-Kendall significance test, and no pronounced step changes were detected, suggesting that vegetation conditions improved persistently and relatively smoothly during the study period. Corresponding to this change, the precipitation also increased significantly over the same period, with a slope of 52.96 mm per decade (Fig. B1), which provides more favorable moisture conditions for vegetation growth. Near-surface air temperature also trended upward (0.17 °C per decade), while this warming was not statistically significant (*p* = 0.114), suggesting that its thermal effect is relatively weak.

**Fig. 7:**
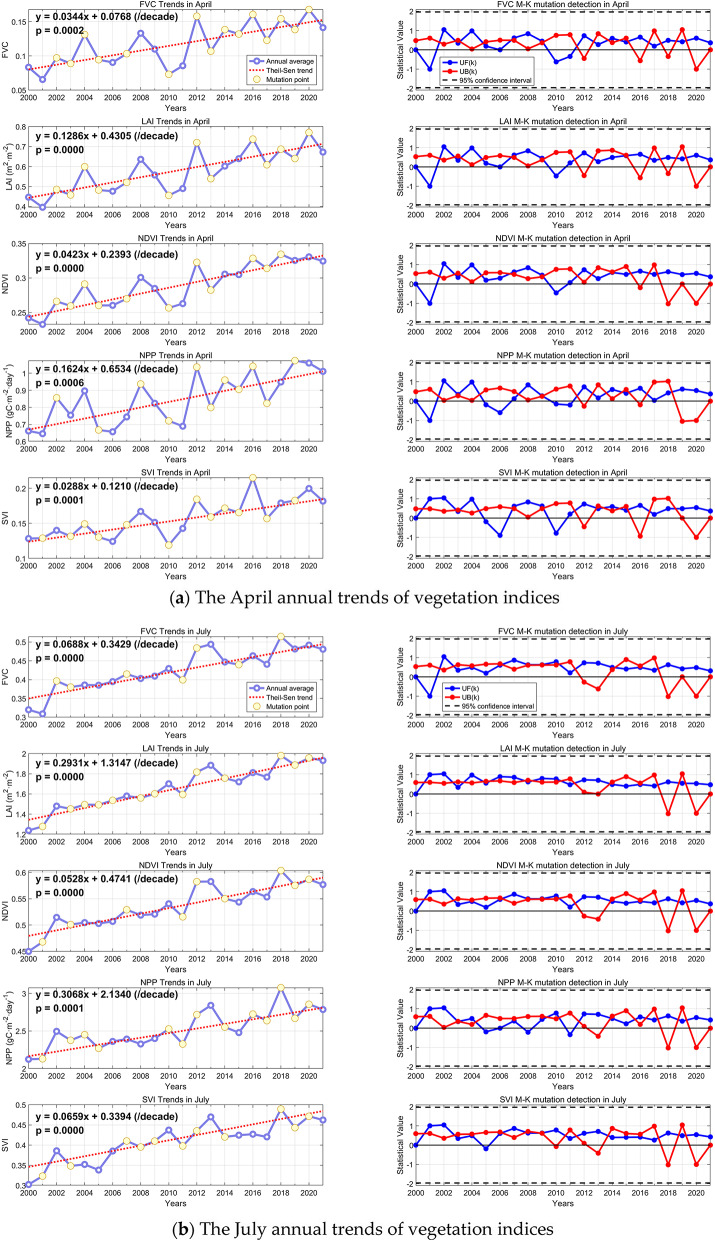

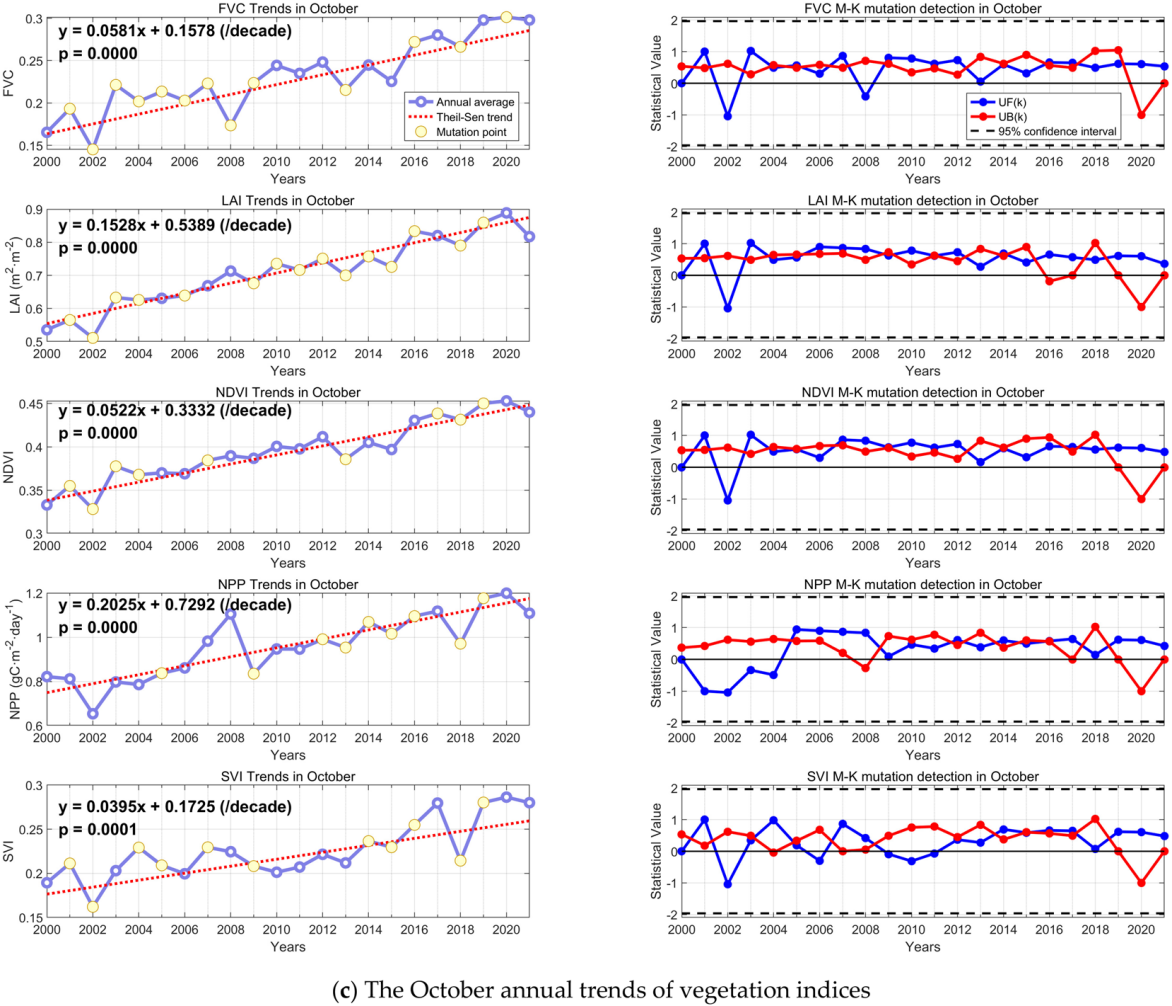
Seasonal mean and trends of different vegetation indices in yellow river basin. (**a**) The April annual trends of vegetation indices, (**b**) The July annual trends of vegetation indices, (**c**) The October annual trends of vegetation indices.

The interannual fluctuations of each index cannot be ignored either. However, the SVI demonstrates strong integrative capacity in representing the composite changes of multiple vegetation indictors. For example, from 2000 to 2001, FVC declined slightly, whereas LAI and NDVI rose and NPP changed little. However, the SVI still showed an upward trend, indicating that it can capture the dominant growth signals better than a single indicator. A similar pattern occurred during 2007–2008, further confirming the robustness of the SVI in dealing with short-term abnormal fluctuations. Conversely, from 2002 to 2003, although FVC, LAI, and NDVI increased modestly, NPP declined noticeably, and SVI decreased accordingly. This highlights that, even if the vegetation structure and greenness improve, the decline in productivity may still suppress the overall vitality of the ecosystem. Furthermore, we observed that between 2002 and 2004, the variation pattern of SVI differed from that of other parameters (e.g., FVC, LAI, NPP). While the latter generally showed higher values in 2004 than in 2002, SVI was lower in 2004. Climatic analysis revealed that this likely resulted from extreme fluctuations in climatic conditions—specifically, anomalously high precipitation and sharply decreased temperatures in 2003. The SVI appears to respond more sensitively to such short-term climatic stressors. This characteristic is further illustrated in the seasonal analysis presented in Fig. 7. During the peak growing season of summer, all indices except LAI, including SVI, exhibited higher values in 2002 than in 2004. Given the dominant role of summer vegetation growth in the annual cycle of the Yellow River Basin, variations in indices during this period carry greater ecological significance. Finally, independent validation using aboveground biomass data confirmed that the dynamics captured by SVI under such climatic fluctuations are more reliable.

#### Seasonal trend

To further analyze the seasonal characteristics of vegetation growth, we analyzed the seasonal trend during 2000–2021. By combining the M-K trend test and Theil-Sen slope estimation to quantify the long-term change rate, and using the M-K abrupt change point detection to identify structural transitions in specific years (Fig. 7). Overall, all vegetation indices show positive trends in different season, with clear differences in magnitude and seasonal distribution. The YRB has a typical temperate continental climate, the mean cumulative precipitation in July is about 80–140 mm and the mean air temperature is 19–21 °C (Fig. B3). From 2000 to 2021, the precipitation in July increased slightly (6.43 mm per decade), while the temperature decreased marginally (− 0.26 °C per decade). Both of these trends have no statistically significant (*p* > 0.05) and exhibits pronounced interannual variability, indicating that there is a strong interannual variability in water and heat supply. Vegetation growth reaches its peak in this season and the changes are in sync with the fluctuations in climatic conditions. The LAI, NPP, FVC and NDVI increased at approximately 0.293 m^2^ m^−2^ decade^−1^, 0.307 g C m^−2^ d^−1^ decade^−1^, 0.069 and 0.053 decade^−1^, respectively. The growth of each index has passed the significance test and significantly exceeded its annual average trend. This suggests that relatively stable or slightly declining midsummer temperatures may alleviate heat stress, and together with weak moistening, helping vegetation maintain its growth vitality.

By contrast, the growth rate during the spring is relatively weak. During April, LAI increases at about 0.129 m^2^·m^−2^ per decade, NPP at about 0.162 gC·m^−2^·day^−1^ per decade, NDVI at about 0.042 per decade, and FVC at less than 0.035 per decade. All of these values were significantly lower than their annual average growth rates. Our analysis shows that the precipitation in April increased significantly during 2000–2021 (+ 0.673 mm per decade), while the temperature showed an upward trend (0.48 °C per decade) but without statistical significance (Fig. B2). This indicates that the soil moisture and temperature conditions gradually improved in the early growth season, which is conducive to the germination and early growth of vegetation. When the vegetation enters the senescence stage in October, the change rates of various indices are similar to those in spring and summer. The growth rates of LAI, NPP, FVC and NDVI are approximately 0.153 m^2^·m^−2^, 0.203 gC·m^−2^·day^−1^, 0.058, and 0.052 per decade, respectively. the mild warming at the end of the growing season (0.18 °C per decade) and the generally stable water conditions (average trend of about 2.5 mm per decade) jointly promoted the delay of the phenological termination period and the continuation of late photosynthesis. Consequently, moderate positive trends are maintained in both structural and functional dimensions of vegetation, which is consistent with the documented “growing-season extension” across North–Northwest China in recent decades^43^.

The mutation points show that different vegetation indices exhibited some common responses yet al.so demonstrate distinct variations. In July, LAI and NPP exhibit the most frequent mutation points, concentrated in 2001–2006, 2008–2012, and 2016–2020, indicating their high sensitivity to water and heat changes (Fig. B3). Notably, these mutation points are not statistically significant. It implies that the observed swings likely reflect interannual climate variability or short-term human disturbances rather than fundamental reversals in recovery or degradation trajectories. The abrupt change points of FVC and NDVI mainly occur in the years with severe temperature or precipitation fluctuations. For example, there are 7 non-significant abrupt changes in FVC and 9 abrupt changes in NDVI. Conversely, both indices respond weakly to large temperature increases: during 2004–2005, 2009–2010, 2015–2016, and 2016–2017, interannual temperature rises exceeded ~ 1 °C (Fig. B3). However, neither FVC nor NDVI has a mutation point, which may be related to their saturability. In contrast, the distribution of mutation points in April was more dispersed, especially after 2010, with at least two indices experiencing mutation points almost every year. Similarly, they are not significant either. The climate trend plots indicate that both the frequency and amplitude of interannual temperature and precipitation fluctuations increased after 2010, consistent with stage-specific effects on early-spring phenology (Fig. B2). In October, despite weak overall climatic trends, interannual variability remains large, the precipitation and monthly mean temperature differences between adjacent years can exceed 50 mm and 2 °C (Fig. B4). This “weak-trend, strong-variability” background, combined with late-season phenological sensitivity, yields frequent interannual perturbations, highlighting the differences in the sensitivity of each index to abrupt changes under similar climate fluctuations.

As an integrated metric, SVI captures mutation points associated with abrupt temperature and precipitation shifts while effectively damping the influence of single-index mutation points on the overall pattern. SVI exhibits significant positive trends in all three months (by 0.066 per decade in July, 0.039 per decade in October and approximately 0.029 per decade in April), which mirrors the seasonal gradient consistent with the single indices. This indicates that SVI stably captures the common long-term signal of concurrent strengthening in structure (LAI, FVC), greenness (NDVI), and productivity (NPP) across growth stages. In terms of interannual perturbations and mutation points, SVI varies more gently. Although it co-oscillates with the single indices over the same periods, its amplitudes are notably suppressed.

#### The spatial patterns of the trends

**Fig. 8:**
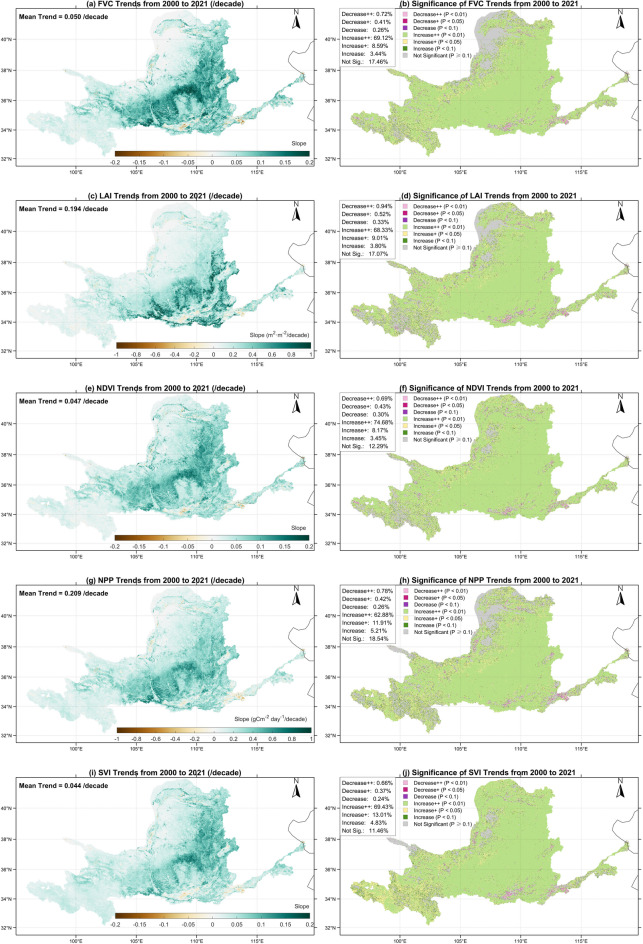
Spatial distribution of average trends and area proportion for different vegetation indices.

Figure 8 shows the spatial patterns of the trends and the seven confidence categories for different vegetation indices. Results indicate strong spatial consistency among indices during 2000–2021, with a basin-wide predominance of positive trends closely linked to overall improvements in hydrothermal conditions across the YRB. NDVI exhibits extremely significant increases (*p* < 0.01) over 74.68% of the basin, substantially higher than the other single indices. While for FVC, LAI, and NPP are 69.12%, 68.33%, and 62.88%, respectively. Areas with the most rapid improvement are concentrated in grasslands of the eastern Loess Plateau and in forested regions of the eastern basin. There, NDVI and FVC increase by more than 0.15 per decade, while LAI and NPP rise by over 0.8 m^2^ m^−2^ and 0.6 g C m^−2^ day^−1^ per decade, respectively. These areas coincide with the focal zones of large-scale ecological programs (e.g., the Three-North Shelterbelt and the Grain-for-Green/Conversion of Cropland to Forest and Grassland initiatives). Springtime warming and moistening elevate the seasonal baseline, and summertime moistening with modest cooling helps alleviate hot–dry stress, together sustaining higher vegetation levels throughout the growing season. In agricultural areas—such as croplands in the southwestern Loess Plateau, along the margins of the Ningxia and Hetao plains, and the North China Plain—all indices also show significant increases, reflecting widespread gains in greenness, canopy structure, and productivity. Beyond climate, this likely relates to advances in agronomic practices, improved water–fertilizer management, and adjustments to cropping systems. Regions with moderately significant (*p* < 0.05) or marginal (*p* < 0.1) increases are mainly distributed in the upper and middle reaches, particularly west of the Hu–Huanyong Line, accounting for roughly 12%–17% of the YRB. These areas are characterized by sparse cover and ecological fragility, with insufficient year-scale co-enhancement of water and heat. Although vegetation conditions show slight improvement overall, strengthened ecological restoration and water-allocation management are still needed.

Because NDVI, LAI, FVC, NPP, and SVI have different units, value ranges, and ecological meanings, their slope magnitudes should not be directly interpreted as absolute evidence that one indicator is superior to another. Therefore, in this study, we focus mainly on the direction of change, significance level, spatial consistency, and local divergence among indicators rather than on direct numerical comparison of slopes. Compared with individual indices, SVI retains the dominant basin-wide greening signal, because SVI is constructed from vegetation-related variables and is designed to summarize their common signal rather than to produce an unrelated spatial pattern. The area fraction with an extremely significant increase (*p* < 0.01) accounts for 69.43% of the basin for SVI, which is lower than that of NDVI (74.68%) and close to those of FVC (69.12%), LAI (68.33%), and NPP (62.88%). This result suggests that SVI provides a more conservative integrated assessment than NDVI alone while still preserving the overall vegetation recovery signal. In areas with dense vegetation cover, such as forests on the Loess Plateau and in the Qinling Mountains, SVI aligns most closely with LAI and NPP, indicating that it effectively reflects concurrent changes in productivity and canopy structure. At the same time, SVI reveals trend discrepancies relative to individual indices in some locations. For example, in zones of cropland expansion or frequent adjustments to seasonal cropping systems (e.g., the western North China Plain and the southern fringe of the Loess Plateau), NDVI and FVC often display marked increases due to rapid phenological turnover, whereas NPP does not rise synchronously. It suggests that enhanced greenness at the surface does not necessarily translate into stronger carbon uptake. In these areas, SVI shows a relatively weaker upward trend, indicating that by integrating multiple sources of information it effectively filters “false greening”. This agrees with recent findings^44^ that optical greenness metrics in arid and semi-arid regions tend to overestimate functional gains and require correction with structural and physiological parameters. In the declining trend area, the spatial distribution of SVI and the single index is largely overlapping, and it is concentrated in the downstream urban agglomerations, the fragile ecological transition zone, and some areas in the northeastern part of Qinghai.

**Fig. 9:**
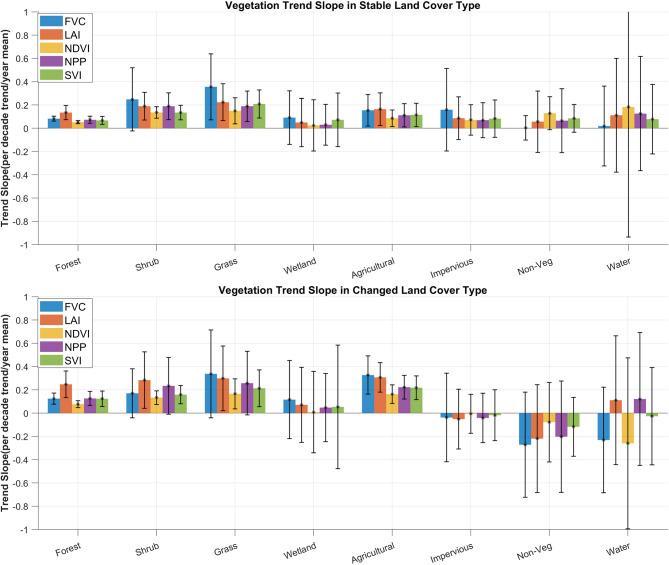
Statistical charts of different vegetation indices changing trends under different land cover.

To further evaluate the applicability and stability of SVI across heterogeneous surfaces, we computed and compared the relative change rate and its standard deviation for five indices (NDVI, LAI, FVC, NPP, and SVI) across eight major land-cover types. Considering the significant land cover transitions from 2000 to 2021, we separately counted the “stable areas” (pixels with unchanged land cover types) and the “transition areas” (pixels that changed from other types to the current type) (Fig. 9). The relative change rate is defined as the decadal trend of each parameter divided by its multi-year mean, which preserves the sign of change while removing unit dependence.

In the “stable” areas, all individual indices exhibit significant positive trends in all natural vegetation classes (forests, grasslands, shrublands, and wetlands). SVI exhibits a relatively high level of relative change rate with notably smaller standard deviations than FVC, LAI, and NPP. In the human-dominated types (croplands and built-up areas), SVI still demonstrates a robust comprehensive characterization ability. However, in non-vegetated classes (bare soil and water-related pixels), FVC trends are near zero, whereas NDVI, LAI, and NPP can show apparent increases. The possible reasons could be that FVC is insensitive to sparse, patchy, or point-based vegetation growth, or that uncertainties and background noise in remote sensing data affect the inversion accuracy of single vegetation index. SVI effectively suppresses such outliers and maintains a small variability. For “transitional” areas, individual indicators often diverge in magnitude because land-cover conversion, seasonal management, or surface background changes may affect greenness, cover, canopy structure, and productivity differently. For example, in croplands and grasslands, FVC or LAI may increase more rapidly than NPP or NDVI, indicating that structural or cover changes do not necessarily correspond to proportional productivity gains. In such cases, SVI rises in concert with the single indices, while its standard deviation is systematically lower than that of NDVI, LAI, and NPP—evidence that SVI reliably captures recovery signals in restoration/expansion zones. Furthermore, in areas converting to built-up, open-water, or otherwise non-vegetated surfaces, negative fluctuations in single indices intensify. SVI, however, tends toward near-zero trends with the smallest variability, indicating strong stability and adaptability even where vegetation signals are weak or atypical. Therefore, the use of SVI provides an intermediate and more conservative signal by integrating complementary information from multiple indicators in heterogeneous or transitional landscapes were relying on a single indicator may lead to incomplete interpretation.

### Independent validation based on AGBD

To further evaluate the reliability of the SVI developed in this study, we employed aboveground biomass density (AGBD) as an independent validation dataset. AGBD, defined as the dry matter mass of vegetation per unit ground area (in megagrams per hectare, Mg/ha), serves as a direct indicator of integrated vegetation growth status and productivity levels, as its spatiotemporal dynamics reflect the cumulative outcome of key ecological processes^45^. Various AGB products have been developed based on data sources such as optical remote sensing, synthetic aperture radar (SAR), and light detection and ranging (LiDAR). This study utilized The China Annual Forest Aboveground Biomass Time-Series Dataset (CFATD) as the benchmark data^46^. CFATD is the first comprehensive, long-term, and high-resolution annual dataset specifically designed for monitoring forest aboveground biomass across China. It provides annual AGBD estimates at a 30-meter spatial resolution from 1985 to 2023. To enhance the accuracy of pixel-wise matching, the China Annual Tree Cover Dataset (CATCD) was concurrently adopted to identify forested areas^47^, ensuring that the analyzed pixels represent typical forest vegetation.

**Fig. 10:**
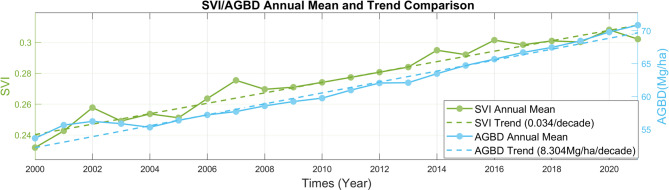
Cross-comparison of temporal dynamics between estimated SVI and AGBD.

We first compared the inter-annual trends of the SVI and the annual mean AGBD from 2000 to 2021(Fig. 10). The results indicate that their inter-annual variations were highly synchronized, with both showing a significant upward trend. The annual mean AGBD increased consistently over the 22-year period at a rate of 8.304 Mg/ha per decade, rising from 53.66 Mg/ha in 2000 to 70.849 Mg/ha in 2021. Concurrently, the SVI increased from 0.232 to 0.302, exhibiting a trend of 0.034 per decade. Notably, during typical climatic anomaly periods, such as the extreme precipitation and low-temperature events from 2002 to 2004, both the SVI and AGBD showed synchronous declines. This demonstrates that the SVI can effectively capture the signals of key climatic and ecological processes that influence vegetation growth and biomass accumulation.

**Fig. 11:**
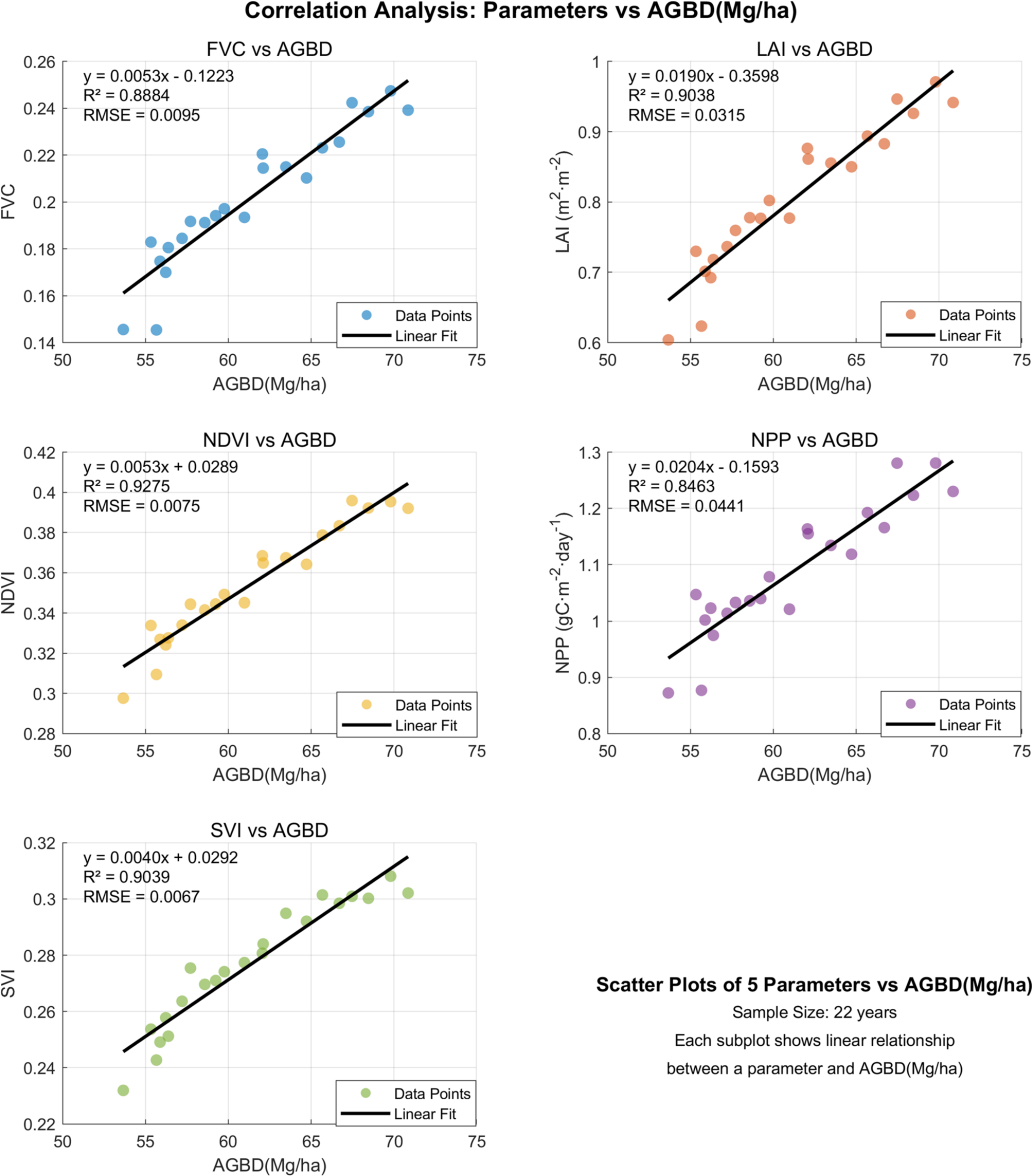
Relationship between annual mean AGBD and different vegetation indictors.

To further quantify the performance of the SVI in characterizing vegetation productivity, we calculated the coefficient of determination (R^2^) and root mean square error (RMSE) between the annual mean AGBD and the SVI, as well as several individual vegetation indices (Fig. 11). Among the five indices compared, the SVI exhibited a strong correlation with AGBD, ranking near the top (R^2^ = 0.9039, RMSE = 0.0067). Its explanatory power was second only to that of NDVI (R^2^ = 0.9275, RMSE = 0.0075) and was significantly superior to the other three indices. It is particularly noteworthy that for sample points where the relationship between AGBD and the vegetation indices deviated from the main trend, the SVI demonstrated greater robustness compared to individual indices, effectively reducing estimation bias. This indicates that the SVI, constructed by using a Dynamic Spatially Variable Weighted approach, provides a more integrated representation of canopy structure, photosynthetic activity, and water status—attributes closely associated with biomass accumulation. Consequently, the SVI establishes a tighter and more stable statistical relationship with AGBD. These results collectively confirm that the proposed SVI possesses higher sensitivity and reliability in reflecting regional vegetation aboveground biomass compared to traditional single indices.

## Discussion

### Advantages and ecological interpretability of SVI

Unlike conventional fixed-weight composite approaches, the DWS framework used the CRITIC objective weighting method to quantify the informational contribution of each vegetation variable every monthly at different land cover, which allows the relative contribution of NDVI, LAI, FVC, and NPP to vary according to local ecological conditions. Where NDVI and FVC convey similar greenness dynamics, LAI or NPP can receive higher weights if they provide additional structural or productivity-related information. Conversely, in sparse or water-limited areas, NDVI and FVC may contribute more strongly because greenness and cover changes are more sensitive. Thus, DWS-SVI is not a simple arithmetic average of existing indicators, but a spatially and seasonally adaptive synthesis of complementary vegetation information^48^.

**Fig. 12:**
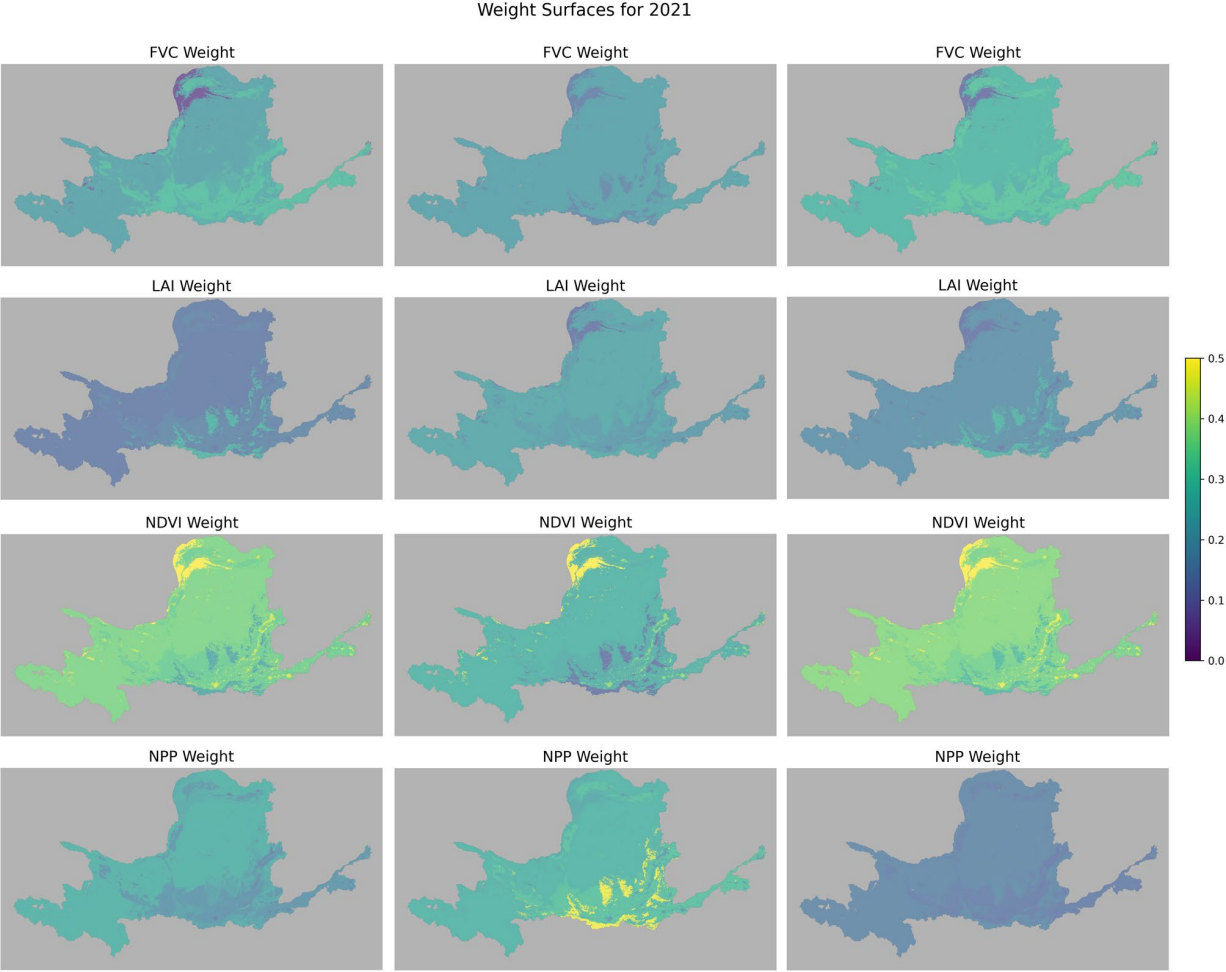
Spatial distribution of the seasonal weight surface in 2021growing season. The panels from left to right correspond to spring, summer, and autumn.

The broad similarity between SVI and the individual indicators is expected, because SVI is constructed from vegetation-related variables, it retains the dominant vegetation-change signal shared by NDVI, LAI, FVC, and NPP. If SVI produced a completely different spatial or temporal pattern from its component indicators, its ecological meaning would be difficult to justify. Therefore, the significance of SVI lies not in producing a different trend, but in providing a more balanced integrated assessment where individual indicators may emphasize different aspects of vegetation condition. In this study, all indicators showed increasing trends, but their ecological implications differ. NDVI mainly reflects greenness, LAI represents canopy structure, FVC describes vegetation cover, and NPP reflects productivity. The SVI trend was consistent with the overall greening signal, while the area with an extremely significant increase (69.43%) was lower than that of NDVI (74.68%) and close to those of FVC (69.12%), LAI (68.33%), and NPP (62.88%). This suggests that SVI preserves the basin-wide recovery signal while avoiding over-reliance on greenness-dominated changes.

To analysis the ecological interpretability of SVI, we produced the weight-surfaces map. Taking 2021 as an example, the map revealed that the proposed DWS–SVI assigns continuous, spatially varying weights that track phenology and local hydro-climatic constraints rather than abrupt changes at land cover boundaries. This demonstrates that our proposed two-step method, which first calculates weights by land cover category and then interpolates them into a continuous weight surface, effectively encodes both categorical differences and landscape gradients into pixel-scale synthesis. This process ensures interpretative consistency and ecological rationality.

In spring, when temperature and soil moisture still limit growth in large parts of the YRB, greenness signals (NDVI, FVC) are preferentially weighted across cropland–grassland mosaics of the southern temperate zone and the Loess Plateau ecotone. However, LAI and NPP carry lower weights. This pattern is consistent with early-season conditions, such as leaf-out and pre-closure canopies, in which structural development and carbon fluxes still lag behind greenness^49^. In semi-arid grasslands of Inner Mongolia–Gansu the dominance of greenness-type weights is further supported by low spring precipitation. Such phase lags between greenness and structure/function are widely documented in cold- and water-limited systems^50,51^. During summer, LAI and NPP weights strengthen basin-wide, particularly over forest belts and irrigated corridors, whereas NDVI weights decline in high-biomass subregions (Fig. 12). This pivot reflects the well-documented NDVI saturation in dense canopies and the superior sensitivity of LAI and productivity proxies to structural/carbon dynamics during peak growth^9,29^. In autumn, with senescence and harvest, NPP weights weaken, FVC/LAI retain moderate importance in agro-forestry areas, and NDVI re-gains relative influence in sparse or partially senescent covers—matching the seasonal shift from productivity to residual greenness^52^.

This seasonal hysteresis mirrors the documented decoupling between greenness and NPP along aridity and management gradients, that is, greenness changes do not necessitate proportional productivity changes^52,53^. Our DWS weights pivoted toward LAI/NPP in high-biomass settings where NDVI saturates, and toward NDVI/FVC in sparse or early-season conditions where LAI and NPP show lower sensitivity to fine canopy variation, which enhances interpretability and guards against “false greening”. Based on these results, DWS-SVI is suitable for integrated vegetation-condition assessment in climatically heterogeneous basins, ecological transition zones and managed landscapes regions.

Furthermore, using GLASS products as inputs strengthens the credibility of the weight patterns and the composite SVI because GLASS LAI/FVC/NDVI (and derived NPP) have undergone extensive cross-product evaluation and direct validation, often outperforming alternative long-term products^24,29^. Thus, the spatially adaptive weighting is built on stable, well-validated inputs, helping ensure that mapped weight gradients reflect ecosystem behavior rather than sensor noise.

**Fig. 13:**
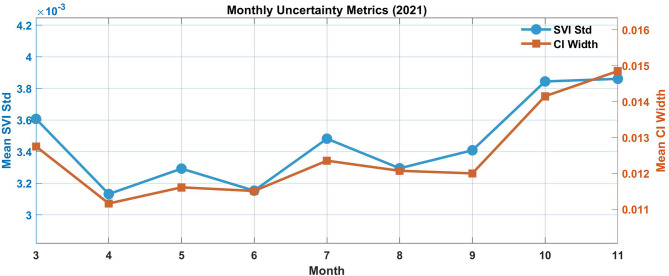
Monthly uncertainty of statistics of the weight perturbation experiment.

### Uncertainty and sensitivity analysis

To assess the robustness of the DWS–SVI, we conducted 100 weight perturbation experiments for the 2021 growing season (March–November). For each month, pixel-level ensembles were generated by randomly perturbing the weights around their baselines, and the standard deviation (Std) together with the 95% confidence interval (CI width) were calculated. These uncertainty metrics were analyzed from seasonal, monthly, ecological, and spatial perspectives to capture the sensitivity of the index to weight uncertainty.

At the seasonal scale (Fig. 13), the SVI exhibited the lowest uncertainty in spring and summer, with basin-mean Std on the order of 10⁻³ and CI widths of ~ 0.012, while autumn showed a clear increase (Std ~ 3.7 × 10⁻³; CI width ~ 0.014). This pattern is consistent with vegetation phenology. In spring, vegetation enters a synchronized stage of greening and leaf expansion, and the coherent development across ecosystem explains the trough in uncertainty observed in April–June. During summer, canopy development and productivity reach its peak, so complementarity information among individual index is sufficient and the disturbances are difficult to amplify. In contrast, autumn is characterized by asynchronous senescence, harvest activities, and management interventions, which amplify the sensitivity of the SVI to weight perturbations. Despite this rise, the absolute magnitudes of uncertainty remain low (Std ~ 10⁻³; CI ~ 10^−2^), highlighting the stability of the index.

**Fig. 14:**
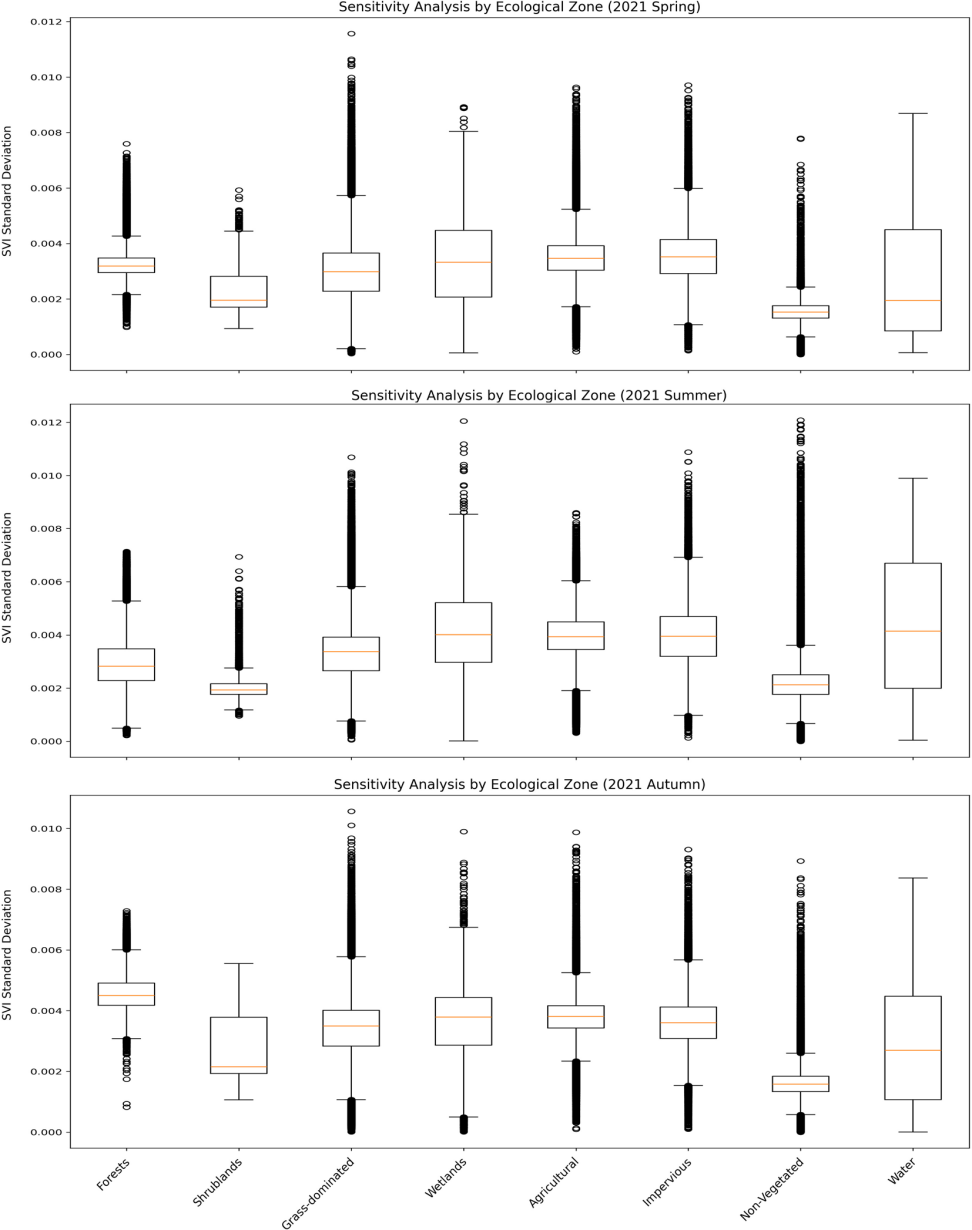
Sensitivity analysis of the weight perturbation experiment across ecological zones.

Ecological zone analysis (Fig. 14) reveals type-specific differences. Wetlands and croplands display the highest sensitivity, consistent with their heterogeneous surface conditions and strong seasonal dynamics driven by water management and land-use practices. Grasslands follow, showing intermediate sensitivity, while forests generally present moderate values but with considerable spatial variability. Shrublands and bare land exhibit the lowest sensitivity, reflecting their relatively simple vegetation cover and more stable weight allocation. Water bodies show a wide-tailed distribution, which is likely influenced by shoreline mixing and seasonal fluctuations in water extent. These patterns are consistent with the DWS weighting logic: allocating greater weight to LAI/NPP in dense vegetation reduces NDVI saturation, while emphasizing NDVI/FVC in sparse or early-season conditions captures subtle greening signals. These results indicate that land-cover heterogeneity is explicitly involved in the DWS-SVI framework rather than being ignored.

**Fig. 15:**
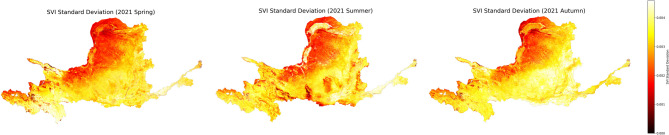
Spatial distribution of uncertainty from the weight perturbation experiment.

The spatial distribution of SVI uncertainty shows a distinct northwest–southeast gradient (Fig. 15). Low values dominate the arid and sparsely vegetated northwest, reflecting the relative stability of weight allocation where vegetation is simple and productivity consistently low. In contrast, higher uncertainty emerges across the more humid and intensively managed southeastern basin, including croplands, wetlands, and ecotonal belts, where complex canopy structures, irrigation, and asynchronous phenology amplify the sensitivity of SVI to weight perturbations. Seasonal comparisons indicate that spring hotspots remain localized in fragmented loess terrain, summer uncertainty is reduced over dense cropland and forest but persists near irrigation zones, while autumn displays the broadest extent as senescence and harvest diversify vegetation states. These patterns demonstrate that spatial heterogeneity, topographic relief, and human disturbance jointly shape the robustness of DWS–SVI, with managed and transitional landscapes requiring greater attention in interpretation and calibration.

### Limitations

Although the DWS–SVI framework substantially improves the reliability and ecological interpretability of vegetation monitoring, several limitations should be recognized. Firstly, DWS-SVI should not be used as a universal replacement for individual indicators. When the research objective is to examine a specific vegetation attribute, such as canopy greenness, leaf area development, fractional cover, or productivity, NDVI, LAI, FVC, or NPP should still be used directly. DWS-SVI is more appropriate when the objective is to evaluate overall vegetation condition or ecosystem functional change, especially when different vegetation attributes may not change synchronously. Secondly, the construction of SVI in this study benefits from the long-term, high-quality GLASS products, which provide a consistent and well-validated foundation for vegetation monitoring. Nevertheless, like all remote sensing datasets, GLASS products may still carry residual uncertainties arising from retrieval algorithms, cloud contamination, or scale mismatches among variables. In particular, NPP is generated through model-based estimation and should not be regarded as an error-free benchmark of ecosystem productivity. In this study, NPP was used only as one component representing the productivity dimension of vegetation condition. Future work should further validate the SVI using independent observations, such as flux-tower carbon fluxes, field biomass measurements, and ecosystem model outputs. Similarly, although MODIS land cover data provide reliable stratification for weight calculation, classification errors may occur in highly heterogeneous or transitional landscapes. In semi-arid and sparsely vegetated areas, soil background and surface albedo may also affect the values of vegetation indicators. DWS-SVI can reduce the influence of single-index bias through dynamic weighting, but it cannot completely remove all uncertainties associated with surface background effects. Incorporating higher-resolution land cover products or data assimilation approaches in future studies would help to refine weight allocation and further enhance the robustness of SVI.

Moreover, although the weighting scheme in this study is objectively determined using the CRITIC method and has proven effective in reflecting local ecological conditions, it currently relies mainly on statistical properties of vegetation indices. This design ensures objectivity and robustness, yet future refinements could benefit from incorporating additional eco-physiological drivers such as soil moisture, vapor pressure deficit, radiation, or land management practices (e.g., irrigation and fertilization). Such integration would not only enrich the ecological interpretability of SVI but also help disentangle the relative roles of climatic and anthropogenic factors. Notably, in special years such as 2012–2013, SVI moved opposite to the common direction of the four indices. Climate data analysis shows that during this period, there was a sudden increase in annual temperature (about 0.6 °C) accompanied by a significant decrease in precipitation (Figure B1). However, the overall decline in vegetation indices in subsequent years may suggest that the SVI exhibits a lagged response in reflecting vegetation growth conditions, which warrants further in-depth investigation.

## Conclusions

This study proposed a Dynamic Geographically Weighted Synthesis Synthetic Vegetation Index (DWS-SVI) for integrated vegetation-condition assessment in climatically heterogeneous basins. DWS-SVI is not a completely new spectral vegetation index, but as a spatially adaptive synthesis indicator that integrates greenness (NDVI), canopy structure (LAI), vegetation cover (FVC), and productivity (NPP) with spatially and temporally adaptive weights. Unlike traditional single indices or fixed-weight combination methods, the DWS–SVI framework allows the relative importance of each indicator to be dynamically adjusted according to land cover types, seasonal changes, and climatic gradients, thereby effectively characterizing ecological heterogeneity. This design not only enhances monitoring robustness by considering ecosystem heterogeneity but also improves ecological interpretability through the matching of weight allocation with the dominant vegetation processes where greenness, structure, cover, and productivity do not change synchronously.

The application to the Yellow River Basin showed that DWS-SVI retained the dominant long-term greening signal detected by individual vegetation indicators and captured clear seasonal vegetation dynamics. Its high-value areas concentrated in the humid southeastern region, while the low-value areas are located in the arid northwestern region, which is highly consistent with the water, heat and topographic constraints. However, it is more sensitive to precipitation. Over the past two decades, more than 69% of the basin experienced significant greening trend, with the strongest improvements in summer, followed by autumn and spring. Compared to a single index, DWS-SVI demonstrates stronger stability and consistency in climatically heterogeneous basins, ecological transition zones and managed landscapes regions where greenness, canopy structure, vegetation cover, and productivity may not change synchronously. The weight perturbation experiment further showed that DWS-SVI had relatively low sensitivity to weight uncertainty, supporting its use as an integrated monitoring tool.

However, this study still has certain limitations. Although DWS-SVI provides a more integrated and conservative interpretation of vegetation condition across heterogeneous landscapes, when the research objective focuses on a single vegetation attribute, such as greenness alone, individual indicators such as NDVI remain more direct and appropriate. Future research should enhance the in-depth analysis of ecological mechanisms and improve the detailed characterization of anthropogenic disturbances. Additionally, it is essential to further integrate eco-physiological drivers, extend long-term uncertainty propagation analysis, and strengthen validation based on ground observations and flux-tower data. These efforts will continuously improve ecosystem monitoring and management capabilities.

## Supplementary Information

Below is the link to the electronic supplementary material.

Supplementary Material 1

## Data Availability

The datasets used and/or analysed during the current study are available from the corresponding author on reasonable request.
