# Supplementary material for: A spatially adaptive synthetic vegetation index for monitoring ecosystem changes in climatically heterogeneous basins
Source: Sci Rep. 2026 Jun 8;16:25946. doi: 10.1038/s41598-026-54880-8 (PMC13486518; doi:10.1038/s41598-026-54880-8)
Supplement: Supplementary file 1 [file 41598_2026_54880_MOESM1_ESM.docx]

Appendix A

The temporal trends of precipitation and near surface temperature in YRB

**Figure A.1.** Annual mean and trends of precipitation and temperature in yellow river basin.

**Figure A.2.** April mean and trends of precipitation and temperature in yellow river basin.

**Figure A.3.** July mean and trends of precipitation and temperature in yellow river basin.

**Figure A.4.** October mean and trends of precipitation and temperature in yellow river basin.

Appendix B

The spatial trends of precipitation and near surface temperature in YRB

**Figure B.1.** The spatial distribution of annual mean and trends of precipitation and temperature in yellow river basin.

**Figure B.2.** The spatial distribution of April mean and trends of precipitation and temperature in yellow river basin.

**Figure B.3.** The spatial distribution of July mean and trends of precipitation and temperature in yellow river basin.

**Figure B.4.** The spatial distribution of October mean and trends of precipitation and temperature in yellow river basin.
